# Role, Resources, and Integration of Accompanying Patients in Oncology: A Qualitative Study from the Accompanying Patient’s Perspective

**DOI:** 10.3390/curroncol33010011

**Published:** 2025-12-24

**Authors:** Sarit Kang-Auger, Margaux Deroi, Khaled Katergi, Soline Bernard, Monica Iliescu Nelea, Cécile Vialaron, Louise Normandin, Marie-Andrée Côté, Mado Desforges, Marie-Pascale Pomey

**Affiliations:** 1 Health Innovation and Evaluation Hub, The University of Montreal Hospital Research Centre, Montréal, QC H2X 0A9, Canada; sarit.kang-auger@umontreal.ca (S.K.-A.); margaux.deroi@umontreal.ca (M.D.); khaled.katergi@umontreal.ca (K.K.); soline.bernard@etu.univ-poitiers.fr (S.B.); monica.iliescu-nelea.chum@ssss.gouv.qc.ca (M.I.N.); cecile.vialaron.chum@ssss.gouv.qc.ca (C.V.); louise.normandin.chum@ssss.gouv.qc.ca (L.N.); marie-andree.cote.chum@ssss.gouv.qc.ca (M.-A.C.); madodesforges@videotron.ca (M.D.); 2 Research Chair on the Evaluation of State-of-the-Art Technology and Methods, University of Montréal, Montréal, QC H2X 0A9, Canada; 3 Poitiers University Hospital Center, 86021 Poitiers, France; 4 Department of Health Policy, Management and Evaluation, School of Public Health, University of Montréal, Montréal, QC H3N 1X9, Canada; 5 Department of Family Medicine and Emergency Medicine, Faculty of Medicine, Montréal, QC H3T 1J4, Canada; 6 Centre of Excellence on Partnership with Patients and the Public, Montréal, QC H2X 0A9, Canada; 7 Support Unit, Learning Health System, University of Sherbrooke, Longueuil, QC J4K 0A8, Canada

**Keywords:** accompanying patient, peer patient, peer support, patient partner, patient expert, oncology, public health

## Abstract

Background: In response to the growing emotional support needs of patients in oncology, peer support was introduced into clinical teams in Quebec, Canada, in 2018. These peers, called accompanying patients (APs), are former cancer patients who use their experiential knowledge to provide support to patients during their oncology journey. This paper aims to identify APs’ perceptions of the program by including a diversity of perspectives. We include inexperienced and experienced APs, APs in different facilities, and APs in different cancer programs. Methods: We conducted a qualitative study based on 12 semi-structured interviews of APs between June and August 2024 in Quebec, Canada. We explored four themes, building on the Practice Change Model for qualitative analysis: APs’ sources of motivation, influences and environmental factors, resources available for AP integration, and the program’s effects. Results: 12 APs from 5 facilities participated in an interview. All the APs, both experienced and inexperienced, were highly motivated to participate in the program. Their motivations included a desire to give back to society, to help people and to give meaning to their illness. Both experienced and inexperienced APs were confident in their ability to accompany others. They were aware of their responsibilities and its limits regarding their role as an AP. They pointed out the program’s positive impact on their own emotional well-being and that of the patients. The program also benefited the clinical team, by limiting unhelpful demands from patients and saving time for clinicians. However, experienced APs did not feel well integrated into the healthcare team. Conclusions: We concluded that APs are highly motivated to be in the program. They perceived a need for the program in the current health system. They noted its beneficial effects on patients, on themselves, and on the clinical team. However, more resources need to be directed toward integrating APs into healthcare teams.

## Simple Summary

Cancer patients face significant emotional and psychological distress during their healthcare journey. To improve care for cancer patients, a peer support program was introduced in Quebec, Canada, in 2018. Former cancer patients are trained to become Accompanying Patients (APs) and then are matched with newly diagnosed cancer patients. APs have unique expertise embedded in their experience with cancer and can orient new patients throughout their treatment and recovery. In this study, we conducted interviews with APs, exploring their motivations, their integration into clinical teams, and the program’s impacts. We found that the APs were highly motivated and confident in their ability to make a difference in patients’ lives and within the clinical team. However, our research highlights how more resources need to be allocated toward integrating APs into healthcare teams.

## 1. Introduction

The number of cancer cases in Quebec has been on the rise for several decades. In 2024, 67,219 new cancer cases were reported, representing 184 per day [1,2]. Despite advanced treatments and reduced cancer mortality over the last few decades, many cancer patients still experience high levels of anxiety and depression [3]. A cancer diagnosis presents psychosocial, organizational, and decisional challenges, severely impacting a patient’s quality of life. Psychological distress is now recognized as a true public health issue. Half of cancer patients are affected by a high level of distress, affecting their well-being and ability to adhere to treatment [2]. However, in the current health system, clinicians lack the time and resources to meet these patients’ emotional needs [4,5]. In response, some healthcare systems have introduced a partnership model [6], in which patients are seen as active agents in their own care. These patients develop experiential knowledge about their disease, its treatment, and navigating the healthcare system [7]. This expertise can be geared toward helping other patients through peer support, by providing valuable informational, emotional, educational and navigational aid.

Several peer support strategies have been tested worldwide to support cancer patients’ emotional health. One strategy introduces patient navigators, who may be nurses, social workers, educators, or former patients. Patient navigators assist patients on a one-on-one basis as they navigate healthcare system barriers [8,9]. Other health systems use support groups, in which cancer patients and/or cancer survivors meet to share their health journey [10,11]. A systematic review of cancer self-help groups found that participants benefited from informational support and shared experience, but group dynamics caused significant challenges [12]. Other types of informal peer-to-peer support programs for cancer survivors have emerged in online settings [13]. Cancer survivors are paired and share their experiences without any facilitation by a health professional. Reviews note that even though these programs have beneficial effects on anxiety, their sustainability would be better assured by concrete cooperation with professional healthcare facilities [13,14]. A structured peer mentoring program has emerged, notably in Vietnam, where breast cancer survivors are matched with newly diagnosed patients with a similar cancer type [15]. Mentors discuss a variety of topics with their mentees, providing advice, and are in close contact with a program coordinator.

Although these programs have a net positive effect on patient well-being [6,7,8,9,10,11,12,13,14,15], many patients’ emotional needs remain globally unmet. In response, the province of Quebec, Canada, launched the research project PAROLE-Onco (The Accompanying Patient, an Organizational Resource as a Lever for an Enhanced Patient Experience in Oncology) [16]. The project introduced Accompanying Patients (APs) within clinical teams. APs are former cancer patients who draw on their experiential knowledge to accompany newly diagnosed patients in their cancer journeys. APs are essential members of the clinical team, as their knowledge complements the team’s expertise. The project is part of a greater transformation of clinical practices in oncology, recognizing the value of APs’ experiential knowledge as a driver of change. It aligns with the work on the co-construction of services and shared governance in health [17].

Since 2019, seven different Quebec healthcare organizations (HCOs) have introduced APs into their teams [18]. APs are recruited on a voluntary basis, without financial remuneration. They attend rigorous training sessions led by several experts, including APs with over three years’ experience. Clinicians offer this service to their patients independently, after which a program coordinator anonymously connects the patient with an AP. Patients and APs conduct meetings by phone or in person. All personal information exchanged is confidential. The number of encounters depends on the patient’s needs.

At the outset of implementing the program for breast cancer patients, our team conducted several qualitative and quantitative studies to assess APs’ impact on Quebec oncology services. These studies showed that APs provide emotional, informational, educational and navigational support to patients. The program also allowed APs to give meaning to their own experience with cancer. They appreciated being able to give back to the community. In addition, APs reduced patients’ anxiety, particularly when the patients had more than one contact with an AP. Finally, limiting factors to AP integration included managers’ and clinicians’ resistance [17].

Since these studies, the program has expanded. This article provides an updated report of its implementation, beyond previous studies of breast cancer APs. Seven facilities introduced the program for five different types of cancer. The seven facilities are CISSS de la Montérégie-Est, Centre Hospitalier de l’Université de Montréal, CIUSSS de l’Estrie, CISSS de la Montérégie-Centre, CISSS de la Côte-Nord, Hôpital Maisonneuve-Rosemont, and Centre Hospitalier de l’Université de Québec. The five cancer programs covered include breast cancer, prostate cancer, lung cancer, colorectal cancer, and lymphomas. The AP program has also been integrated into the Ministry of Health and Social Services’ priority guidelines. The objective of this study is to analyze APs’ perceptions of their role by studying (1) their motivation for participating, (2) the environmental factors influencing the program’s implementation, (3) the resources available for AP integration, and (4) the program’s effects. We include the views of both experienced and newly trained APs. This is the first article to include such a wide variety of perspectives (facilities and programs) on the PAROLE-Onco program.

## 2. Methods

### 2.1. Study Design

We conducted a qualitative study, including semi-structured interviews with the APs conducted between June and August 2024, to shed light on their perspectives on the peer support programs after five years of implementation [16,18].

### 2.2. Participant Selection

The inclusion criteria were (1) being an AP participating in the PAROLE-Onco project, (2) being trained in one of the seven HCOs that have implemented the program, and (3) being able to communicate in French. Two of the HCOs are not represented in this study, as interviews with their APs could not be conducted within the required timeframe. The interviews with these APs will be included in a future study. The AP selection process was developed using a methodology that takes into account criteria for a diversity of experiences: APs who had spent more than two years accompanying patients versus new APs; APs recruited into different cancer programs; and APs recruited in urban, semi-urban and rural HCOs. We included inexperienced APs, as we were interested in exploring their unique perspective on their future role and responsibilities, their motivations, and how the training process impacted them. We classified APs as “experienced” if they had already begun accompanying patients (i.e., had completed at least one accompaniment) and as “inexperienced” if they were newly trained at the time of the interview. A summary of the sample of APs is presented in Table 1.

### 2.3. Data Collection

The interview guide was organized according to a constructive conceptual framework: the Practice Change Model for qualitative analysis [19,20]. It covers sources of motivation for APs, environmental factors, the program’s effects, and the resources available for AP integration (see File S1). The interview was pilot-tested with two researcher-accompanying patients (MAC and MD) to refine the guide and ensure clarity. APs without accompanying experience answered questions based on their personal experience with cancer, their experience during the training, and their perceptions of their future role. The average time spent in the interviews was 30 min. All participants signed a consent form, and the semi-structured interviews were recorded and transcribed. The transcripts were anonymized. The interviews were conducted by telephone and video conference (TEAMS or Zoom) in French by four researchers (KK, MD, SKA, SB). No compensation was offered for participating in an interview.

### 2.4. Data Analysis

To ensure rigorous analysis, we followed the Standards for Reporting Qualitative Research (SRQR) [21]. For data analysis, we used thematic analysis and iterative coding, following five steps: (1) familiarization with the data, (2) generation of initial codes, (3) identification of themes, (4) review of themes, and (5) refinement and definition of final categories [22]. To familiarize themselves with the data, the researchers read and transcribed the interviews. Three researchers (KK, MD, SKA) independently coded the data to enhance reliability. To generate codes and identify themes, they coded the same three interviews, using a combination of deductive and inductive analysis. They used deductive thematic analysis, using the themes described in the Practice Change Model. They used an inductive approach [23] to allow new subcategories to emerge for each theme, building upon the Consolidated Framework for Implementation Research (CFIR) [24]. Researchers held regular meetings to resolve discrepancies and harmonize the scope of each subcategory. Once they had formalized the sub-categories in the codebook, the researchers coded all the interviews using QDA Miner Software version 6.0.2. Two researchers (MD, SKA) separately selected relevant verbatim transcripts from each interview according to each subcategory and then compared them for added rigour. The verbatim transcripts were translated into English and re-translated into French to ensure the accuracy of the translation.

### 2.5. Ethical Considerations

This study was approved by the Centre Hospitalier de l’Université de Montréal (CHUM) Research Ethics Board (REB), which served as the reviewing REB for all participating sites in this multicenter project. All participants provided written informed consent using the REB-approved information and consent form (project ID: 22.155). The form was distributed to participants by email and returned, duly signed, via the same channel in accordance with institutional policies and applicable regulations.

### 2.6. Trustworthiness

We implemented several strategies to enhance the trustworthiness of our qualitative analysis. To strengthen the data’s credibility and dependability, three researchers (MD, SKA, KK) independently coded the transcripts, compared their coding, and discussed discrepancies until consensus was reached, following a systematic five-step thematic analysis process and a shared, iteratively refined codebook. Confirmability was supported by an audit trail documenting coding decisions and theme development. We promoted transferability by using maximum variation sampling (experienced and inexperienced APs, several facilities and cancer programs) and by providing rich descriptions of the study’s context. Lastly, we fostered reflexivity through regular team discussions on how our professional and experiential backgrounds might influence data interpretation, with a constant effort to remain close to participants’ own words when generating themes.

## 3. Results

### 3.1. Participants’ Characteristics

Among the 19 APs contacted, 12 APs agreed to be interviewed (response rate: 63%). Data saturation was reached after these 12 interviews. Data saturation is reached when no new themes emerge during qualitative analysis. Rather, verbatim transcripts confirm previously identified categories. We reached saturation after seven interviews, and five more were conducted. There were 3 APs from F1 (17%), 5 from F2 (42%), 1 from F3 (8%), 1 from F4 (8%), and 2 from F5 (17%) (Table 1). The 5 APs from F2 were involved in the prostate cancer support group. The other APs were part of the colorectal (25%, n = 3), breast (17%, n = 2), or lung (8%, n = 1) cancer programs in their respective facilities. 7 APs had already begun accompanying patients (58%), while 5 were newly trained or still in training (42%). 6 APs were women (50%) and 6 APs were men (50%). Table 2 presents the participants’ characteristics. At the time of data collection, the program was still in an early implementation phase in all the participating facilities; experienced APs had generally been in the role for less than three months and had accompanied no more than a handful of patients (up to approximately five).

### 3.2. Qualitative Analysis

The qualitative results are presented according to four themes: AP sources of motivation, influences and environmental factors, resources available for AP integration, and the program’s effects. For clarity, each of these themes and their subthemes is presented in its own subsection (Section 3.3, Section 3.4, Section 3.5 and Section 3.6), with descriptive subheadings and illustrative quotations integrated directly into the text.

The verbatim transcripts are presented with the facility number and the participant number (see Table 2).

### 3.3. Motivations of APs

This section explores the APs’ motivations for participating in the PAROLE-Onco program. These motivations revolve around their individual characteristics, knowledge, perceptions of self-efficacy, and personal experience with cancer.

#### 3.3.1. Individual Identification with the Organization

No AP specifically stated that identification with their hospital motivated them to participate in the program. However, participants from F2 learned about the program through their hospital-organized cancer support group. Their familiarity with the hospital environment and sense of belonging motivated them to take part in the AP program. Familiarity with the facility could also be important for AP integration; an AP who had not begun accompaniment expected to be easily integrated into the hospital, which was a place she “*knows very well of course*” (F5-01). F5-01 was confident that her familiarity with the facility would facilitate her integration into the health team as an AP. However, she expressed some doubts about communication within the clinical team concerning the AP’s role. No experienced AP mentioned ongoing feelings of identification with the organization throughout their accompaniments.

#### 3.3.2. Knowledge of the Intervention

Several APs expected the program to have beneficial effects on patients, which motivated them to participate. An AP who had not begun accompaniments wanted to “*help others, [whether it be] through volunteering or not*”, to “*be there for the patient, and to listen to them*” (F1-02). APs who had already begun accompaniments were confident in the positive impacts of the intervention. F3-01 had faith in the intervention, while F2-01 expected to “*bring hope to patients*”. F2-01 also mentioned that the program could be “*complementary to the prostate support group*”. Another AP wanted to soothe patients’ fears “*because the unknown is in fact the scariest aspect of cancer*” (F4-01).

#### 3.3.3. Self-Efficacy

Self-efficacy is the belief in one’s capacity to reach certain goals. Newly trained APs were already confident that they would be able to help patients, which would be gratifying.

“*I’ve always known I would help others*”.

(F1-02)

“*It’s gratifying to know that we will be able to help others*”.

(F2-02)

F2-02 was ready for an ongoing learning curve to improve their capabilities: “*Feedback is very good, so we can learn, and as we help, we learn [and improve] even more*”. APs who had already begun accompaniments were similarly confident in their ability to have a positive impact on patients.

“*When I finish a call, […] I know that I have made a difference for the patient*”

(F3-01).

“*It’s very satisfying and gratifying*”

(F4-01).

However, some expressed self-doubt, mainly because they were matched with a patient whose treatments differed from theirs. “*I could advise a patient [on the ostomy I had], but I haven’t yet had a patient with an ostomy*” (F1-01).

#### 3.3.4. Personal Experience

Almost all the APs interviewed spoke about their past patient experience as their main motivator, whether they had begun accompanying or not. Many believed that they would have benefited from the program themselves. An AP who had not yet begun accompaniments said, “*I would have been happy to have someone myself during [my medical journey]*” (F1-02), while an experienced AP said, “*I didn’t have the chance to be accompanied when I had my surgery*” (F4-01). Other APs benefited from informal accompaniments during their cancer treatments, which was a motivating experience. An experienced AP said

“*Why am I doing this? It was very beneficial to talk with someone who had had a similar experience four years before, and to see that the patient was still functional today. It gave me hope*”

(F2-01).

Many patients, like F5-02 who had not yet begun accompaniments, wanted to “*give back to the community*”. Other APs thought the program would be cathartic. An experienced AP said, “*I wanted to give meaning to what I myself had experienced*” (F3-01).

### 3.4. Outside Motivators

Outside motivators refer to the external influences and environmental factors that encourage the implementation of an AP program.

#### 3.4.1. Patient Needs

Patients are in need of greater psychosocial support, and the PAROLE-Onco program is a good response. APs who had begun accompaniments stated

“*Patients are anxious because they don’t know the treatments, they don’t know the side effects*”

(F2-04).

“*The pre-diagnosis period is very stressful. Patients need to have time to share their concerns, reflect, and be listened to*”

(F3-01).

APs provide much-needed psychosocial support. They create much-needed space for patients to decompress, reducing anxiety:

“*The patient can express himself more easily when he’s with someone like him. He identifies [with the AP]*”

(F2-03).

APs also help patients formulate questions to ask their doctors. A newly trained AP explained that many patients need help understanding medical terms. These misunderstandings prevent patients from asking questions: “*It’s either a language problem or a comprehension problem*” (F2-05). F2-05 also wondered if “*there could be big differences between the attitudes [and needs] of men and women. Men might be less inclined to share their experience and speak about it*” (F2-05).

#### 3.4.2. Peer Pressure

Since the AP program is new, it is not widespread across Quebec HCOs. However, HCOs with well-established programs could leverage a form of “peer pressure”, fostering AP integration in newer facilities. Some APs with newer facilities and programs mentioned being inspired by the large breast cancer program in F2. “*We want to get inspired by the breast cancer program, a program I respect. I admire them a lot*” (F2-04). However, peer pressure could be counterproductive. Some APs expressed discouragement when they compared the lack of resources in their hospital to that in larger hospitals:

“*Other hospitals already have well-established programs, like in F2, and they can go further with their APs. Since we’re just starting out, it’s a bit difficult*”

(F1-01).

“*In F2, PAROLE-Onco is well established, so there are a lot of APs with various cancers. For us, I’m the first AP, and we have so much to do*”

(F4-01).

Another AP mentioned that seeing the well-established program in F2 motivated them to “*promote the program a bit more*” (F5-02). However, an AP from F3 was hesitant to compare their hospital’s program to F2′s, saying that regional differences need to be taken into account in program implementation: “*Each region has to give their own flavor [to the program]. We are different from F2. Our approach has to be different*” (F3-01).

#### 3.4.3. Culture

Culture refers to the way the hospital environment encourages or discourages the program’s implementation. One important catalyst is the appreciation for APs among hospital workers. Many APs did not feel valued by the clinical team. The APs understood that “*doctors, and nurses,* etc.” are part of a different world, which affects “*appreciation of APs*” (F4-01). Several APs hoped that the program coordinator could promote the AP’s role to the clinical team, legitimizing the project. “*As soon as the head of the department believes in the program, things will advance more quickly*” (F2-02). F2-02 also wished to be informed of the progress made in PAROLE-Onco research projects, which would further foster integration and appreciation: “*It’s important to know how the research project is going. It gives us strength*”.

#### 3.4.4. Structural Origins of the Intervention

Structural origins are aspects of the current health system that reveal a need for the new AP program. One of these structural origins is health professionals’ lack of time to adequately meet their patients’ needs. Almost every AP interviewed emphasized this:

“*The physician doesn’t have the time [to answer questions and respond to the emotional needs of the patient], and neither does the nurse*”

(F2-03).

“*In some departments, everything goes so quickly*”

(F4-01).

An AP who had already begun interventions noted that APs can fill this gap, since they have more flexibility in the time they can spend: “*Everything that you don’t have time to speak about with your doctor or nurse, you can speak about with the AP*” (F4-01).

Some APs mentioned their retirement as a factor that gave them greater availability.

“*Most of the APs are retired. So, they have a bit more time*”

(F2-01).

“*I’m retired, so the program gives me a project where I have a role to play*”

(F3-01).

### 3.5. Resources Available for the Implementation of the New Program

This section describes the factors that facilitate or limit implementation of the AP program.

#### 3.5.1. Structural Characteristics

The integration of APs into their medical center and clinical team requires organizational resources. Many APs expected meetings with the clinical team, which could improve communication. However, such meetings were rare in all the facilities. “*The link should be tight between the clinical team [and the AP], but this link was insufficient*” (F2-04). In F1, the APs had meetings to talk in a community of practice. An experienced AP expressed that those regular meetings between APs enabled them to be “*used to talking with each other*” (F1-01). Another experienced AP identified institutional email addresses as structural barriers to their integration: “*I started with my personal email, but it isn’t as professional as my [institutional email] right now*” (F3-01).

#### 3.5.2. Network and Communication

Many APs reported a lack of communication with the clinical team. This may be because the program is in its early stages on several sites. An AP who had not begun accompaniments already perceived a lack of communication, stressing the importance of “*properly explaining [the AP] role to the medical team*” (F2-05). An experienced AP similarly mentioned:

“*I don’t yet feel integrated into the clinical team, but that’s something I want, even if we don’t need to communicate often. It isn’t easy to get access to the team. This communication could be something that would help the clinical team provide better treatment*”

(F4-01).

In addition, one experienced AP mentioned that “*it wasn’t always useful to communicate with the clinical team*” (F1-01). This AP mainly communicated with the program manager at their site. An AP from the same site also stated, “*When APs do have contact, it is mainly with the nurse coordinator*” (F1-03). Overall, the APs said they needed greater communication to foster their integration into the clinical team.

#### 3.5.3. Role of the Program Coordinator

Some APs emphasized the importance of the role played by program coordinators in improving communication with the clinical team: “*Our coordinator works in the hospital, conveying important information to the AP and modifications to procedures. They give us good guidance*” (F1-02).

The coordinator is key for following up with APs after their interventions, especially since “*the clinical team is always so busy*” (F4-01). F1-03 mentioned that the program coordinators can facilitate the integration of APs into the clinical team as they are established members of the clinical team: “*The program coordinator is the link between the AP and the clinical team, since she works in the hospital*” (F1-03). Accordingly, they can legitimize the APs’ role to the health professionals. Another AP used the term “champion” to describe their program coordinator: “*to achieve this integration, the champion has a lot to do*” (F3-01).

#### 3.5.4. Characteristics of APs

Certain AP characteristics influence the program’s implementation. The characteristics that participants recurrently identified were “*listening skills, communication skills, and empathy*” (F4-01). The role of an AP is “*to be an ear, [...] an echo chamber for the person*” (F1-03).

APs also recognized the significance of their experiential knowledge. They are the only members of the health team able to say, “*I understand what you are going through because I lived through it myself*” (F5-01). Empathy created a distinct relationship of trust between APs and patients:

“*From the minute we talk about our experience, what we went through, that we went through the same things as the patients, there is already a bond created*”

(F4-01).

However, APs are aware that the AP–patient connection should remain professional. An AP who had not begun accompaniments expected that professionalism would “*control the emotional aspect*” of the intervention (F2-02). The APs knew the limits of their role:

“*We can speak about our experience. […] We’re not there to give the patient answers. We can’t say what the doctor will decide to do, but we can refer the patient’s medical questions to the nurse*”

(F1-03).

APs also identified availability as one of their key characteristics: “*That’s what we have, we’re available for the patient, with time to talk*” (F4-01).

#### 3.5.5. Tools Used

Many tools were used to implement the program. The APs felt that they were sufficiently trained by the online modules. APs and patients communicated by telephone, with hidden caller ID for the APs. All the APs, both experienced and new, accepted the telephone as a proper mode of communication: “*It allows us to keep our privacy. […] By telephone as well, our listening ability increased. There are a lot of benefits*” (F1-02).

F1-03 appreciated the anonymity that the telephone offered, especially during first contact with a patient. Conversely, another AP highlighted the benefits of video calls. “*Seeing the non-verbal side, hand gestures, the face helped the AP better assess the patient’s situation*” (F2-02). Overall, phone or video communication fostered professionalism and ensured confidentiality between APs and patients.

### 3.6. Effects of the Intervention and Opportunities for Change

This section describes how the program impacts patients, APs, and clinical teams. It also includes suggestions for improving the program.

#### 3.6.1. Effects on the Patient

APs who had started to follow patients perceived that accompaniment improved patient well-being and reduced patient anxiety. Patients “*were very thankful*” for the AP (F3-01). Within the overwhelmed health system, “*patient accompaniment helps interactions remain more human*” (F3-01). This humanization is in response to the “*depersonalization of the entire medical system*” (F1-03). An experienced AP foresaw an improvement in information literacy: “*It’s definitely going to be good for the patient, [...] because when I was alone with my Internet, it was telling me all kinds of horror stories*” (F2-03).

Similarly, APs who had not begun accompaniments predicted that they would reduce “*a form of nervousness [among patients]*” (F2-02) as well as “*give them hope*” (F1-02). Another inexperienced AP added “*I think that to have the ear of someone who had taken that path is exactly what could be beneficial for the person to be accompanied*” (F5-02).

#### 3.6.2. Effects on the AP

APs who had started accompaniments explained that the program helped them pursue their cancer healing journey: “*It gives meaning to what we have lived through*” (F3-01). They felt a sense of accomplishment: “*It may sound selfish, but we feel good helping others*” (F2-03). An AP who had not started accompaniments added “*Because I’m not capable of doing anything physically, [...] I have this kind of satisfaction to be able to help people with their problems*” (F2-02).

Other APs who had not started accompaniment faced psychological challenges during their training:

“*It forced me to relive what I had gone through ten years ago, which was a bizarre feeling*”

(F1-02).

“*It could become very emotional, because we’re conscious of what others are going through. We’ll have to keep a certain distance from that*”

(F5-02).

#### 3.6.3. Effects on the Clinical Team

Experienced APs impacted the clinical team by bringing “*additional information*” about the patient to the team (F2-03) and by reducing the “*education [burden on health professionals]*” (F3-01). “*We can bring another perspective*”, F2-03 added. One patient partner also mentioned that the explicit recognition of their lived experience by healthcare professionals strengthened their sense of legitimacy.

“*With collaboration, I feel like I’m a part of the clinical team. And I know that the pharmacists in F3 appreciate my presence*”

(F3-01).

APs also improved morale on the team; an experienced AP perceived that “*when you feel you’ve made a difference in a patient’s life, it’s good for the morale, even in the hospital*” (F2-01). APs even helped the team become more efficient. Stressed patients turned to APs rather than a doctor, which prevented “*unnecessary calls to the professionals*” (F4-01).

“*Physicians are more efficient in their meetings with patients, who are better informed thanks to the time we spent with them*”

(F4-01).

#### 3.6.4. Suggestions to Improve the Program

APs had many suggestions to help implement the program. Newly trained APs suggested regular discussion groups for APs, for conversation, giving advice to new APs, and having space to “*let off steam*” (F5-02). F5-02 also suggested having “*refresher sessions*” on training materials. F2-05, who was also newly trained, stressed the importance of better informing the clinical team about the role played by APs. This would make clinicians “*more comfortable referring patients to APs*” (F2-05).

Clarification of the AP role could also help promote the program, which had not been implemented at certain sites (F5-02). Furthermore, many experienced APs wanted to improve their communication with the clinical team. The team would benefit from “*feedback from the accompanying patients*” (F4-01). To optimize support, another experienced AP suggested beginning to follow patients from “*the moment they learn they have cancer*” (F1-01).

Many experienced APs emphasized the benefits of following a patient with the same cancer and treatment trajectory as their own. F1-01 further suggested offering to match patients and APs by gender. Overall, the APs had many suggestions on how to improve the program, but one AP was disappointed in the length of the process: “*We need to [take action]. It’s been a year [...] and nothing is happening*” (F2-03). Table 3 summarizes the factors limiting AP integration and recommendations on improving it.

## 4. Discussion

Since the PAROLE-Onco program has been launched in multiple new healthcare organizations in Quebec, our objective was to analyze Accompanying Patients’ thoughts on their motivations and integration, and the program’s effects. As some hospitals have had few accompaniments, our study is one of the first to include data from both experienced and inexperienced APs. We have also included the perspectives of APs from various facilities and cancer programs.

### 4.1. Motivations of APs

Stakeholder motivations are crucial for implementing change in healthcare [19], and our study explicitly investigates APs’ motivations. The APs were highly motivated to volunteer, whether they had started accompaniments or not. They were motivated for various reasons, such as improving patient well-being. Before beginning accompaniments, the APs were already confident in their ability to make a difference for patients. Past literature corroborates the finding that gains in self-esteem contribute to continued motivation among volunteers [25]. In our study, experienced APs felt gratified by the hope they brought to new cancer patients, which continuously motivated them. In our team’s 2023 interviews, APs similarly mentioned feeling that the intervention was personally rewarding [17]. We further brought to light that this feeling could be an essential source of motivation, even before beginning accompaniments. Another source of motivation could stem from the organizations themselves. Studies on senior and adult volunteering found that being supported by their organization is highly motivating for volunteers [25,26]. In our study, though many participants did not feel adequately supported by their HCO, familiarity with their facility was encouraging for some. Generativity and life experience can also motivate volunteers, as analyzed by Yamashita et al. [27]. They specified that motivations to volunteer vary across life stages, and generativity is an especially significant motivator in the later adulthood group. We did not assess age in our study; however, almost all the APs stated that they were strongly motivated to volunteer by their personal experience with cancer. Myrhoj et al. further studied catharsis as a motivator for volunteers [28]. They found that among leukemia peer supporters, recovered patients used volunteering to bring cathartic meaning to their own cancer experiences. Newly trained APs similarly expected the program to make their illness meaningful. Previous studies certify the therapeutic effects of peer support, as patient volunteers report personal growth, emotional resilience, and a reinforced sense of purpose as key outcomes of their involvement [29].

### 4.2. Outside Motivators

Several aspects of the current Quebec health system point to a need for the AP program. APs noted that social and emotional support for cancer patients is currently lacking, despite the greater attention given to mental health in recent years [3]. Empathy among physicians may be one method to improve emotional support. A 2023 study found a high association between physician empathy and cancer patient outcomes [30]. Several other studies have found that empathy contributes to trust in the physician–patient relationship [7,31,32]. In our study, the APs at all HCOs and in all cancer programs reported that physicians do not have sufficient time to respond to patient needs by showing empathy. Studies of various healthcare systems highlight time management as a particular challenge for physicians, primarily due to excessive pressure and responsibilities [4,5]. In contrast, APs’ greater availability allows them to show empathy to patients. In a past study of the program from the patient’s perspective, interviewees expressed that the AP’s availability allowed them to share their emotions [17]. Our study suggests that, as the program and the health system evolve, time and empathy remain key aspects of the contribution made by APs.

Within the evolving patient-physician relationship, patient expertise has gained greater value over the past few years. In many healthcare systems, patients are now involved in shared decision-making regarding their treatment options [6,7]. However, a 2021 review found a mismatch between patients’ desired involvement in their cancer treatment decisions and what they actually experienced [33]. APs may be the missing link between patients and care providers, as they encourage shared decision-making. According to our findings, APs’ lived experiences give them a type of expertise that complements clinical expertise. This unique positioning enables them to build trust with patients, providing both informational and emotional support. Further research is needed to explore how APs might improve patient outcomes, particularly by encouraging treatment adherence and reducing decisional regret.

### 4.3. Effects of the Intervention

Previous literature on the role of peer support in oncology emphasizes its impact on patient empowerment and emotional resilience [12,34]. Ziegler et al. state that peer support should be more systematically valued in cancer care, as it is empowering [34]. In this study, all interviewed participants reported positive effects of the intervention on patients, many of whom expressed gratitude toward APs. Thanks to their shared cancer-related experience, many APs fostered a unique form of care for their patients. These findings complement our team’s past interviews with APs, nurses and the patients themselves. In these studies, APs contributed to decreased anxiety among patients [16,17]. This decrease allowed the patients to retain more information and pose more reflective questions during meetings with their physician. APs also helped patients navigate the health system, building their confidence to become active agents in their care. The present study adds that even before beginning accompaniments, the APs perceived the program’s positive effects on patient well-being. It may thus be crucial to emphasize the program’s beneficial effects during training sessions, as it empowers new APs. APs even helped build trust within the physician–patient relationship, which has been shown to impact patient outcomes [34,35]. Patients from marginalized groups could benefit from this increase in trust. Studies show that patients from minority groups face cultural barriers that lead to distrust in the physician–patient relationship [35]. Furthermore, these negative relationships are associated with increased mortality, contributing to health inequalities. Further research is needed to investigate how APs may mend the relationship between physicians and patients from minority groups.

Our study reveals that the program had a positive impact on the APs themselves. Newly trained APs already felt this positive impact, even before beginning accompaniments. The literature has long noted that patient volunteering is cathartic [36,37,38]. Daily participation in volunteer activities may even decrease depression among the elderly [36]. The APs we interviewed were glad to see the effects of their engagement on their own well-being. In a past study that interviewed APs in breast cancer programs, participants gradually grew more confident in their accompanying abilities, resulting in feelings of reward and purpose [17]. However, the effects may not all be positive. In our study, a newly trained AP mentioned that it felt odd to relive their past cancer experience. An AP interviewed in a past study similarly mentioned that it could be emotionally burdening to listen to some patients’ cancer stories. Volunteering may help such patients develop abilities to cope with their life situation [39]. Nevertheless, our findings suggest that it would be wise to include resources in the training sessions to mitigate the risk of emotional burden. Further studies are needed to assess the long-term psychological effects of sustained engagement as an AP.

As the program grows, so do its effects on the clinical team. As previously mentioned, APs increase physician empathy, which strengthens the physician–patient bond. APs can even transfer their experiential knowledge to clinicians, helping them foster a better understanding of the non-medical needs of their patients. The dialogue between different forms of knowledge can thus improve patient care. The APs in our study also believed that the program reduced physicians’ workload. Breast cancer APs interviewed in 2023 similarly explained that APs shared navigational information with patients, which helped save time in subsequent meetings with professionals. APs from our study further added that this increased efficiency could improve team morale. To our knowledge, no studies have identified the effect of APs on team morale. Further studies are needed to investigate attitude changes in the clinical team once an AP has been added. However, these positive effects may only become apparent in hospitals with a well-established AP program. Thus, efforts should be made to ensure proper AP integration.

### 4.4. Resources Available for the Implementation of the New Program

Despite efforts to improve AP integration into clinical teams since the program began in 2019, our results indicate that APs remain insufficiently integrated. Our team’s past studies found similar difficulties [16,17], emphasizing the challenges inherent in introducing a new program into an ingrained health system. Few reports in the scientific literature analyze ways to integrate peers into clinical teams. Grim et al. state that organizational issues prevent peer supporters from providing support to their full capacity [40]. They mention that hospital managers have significant influence on program implementation, but their lack of knowledge about peer supporter roles hampers integration. One AP interviewed mentioned that health professionals could promote the legitimacy of APs within the clinical team. In our team’s 2024 study on AP integration, several cultural and personal barriers prevented health professionals from supporting the program [18]. One such barrier was ego, which led some physicians to feel that they were already fulfilling the AP’s role themselves. Other limiting factors related to misunderstandings regarding the role. Some health professionals were unsure whether APs would recognize the ethical boundaries of their role. For instance, they thought APs might give erroneous clinical information to patients, since they have no medical expertise. However, in our study, all the APs took care in delineating the boundaries of their role. Thus, some misunderstandings may originate from external stakeholders rather than the APs themselves. To correct these misunderstandings, the APs suggested arranging meetings among APs, as well as meetings with the clinical team. Englander et al. provide comprehensive recommendations on how to integrate peer mentors into hospital settings [41]. They emphasize the importance of clearly defining the peer role, including peers in regular team meetings, and introducing peers to staff through flyers and newsletters.

Since the AP role lacks formal institutional acknowledgment, it is not consistently accepted in clinical teams. Reinforcing institutional acknowledgment would increase APs’ legitimacy and, consequently, their integration. In 2018, Quebec’s Health and Social Services Department developed a framework for the partnership approach involving service users, their families, and health and social services stakeholders: the “*Cadre de référence de l’approche de partenariat entre les usagers, leurs proches et les acteurs en santé et en services sociaux*” [42]. This framework defines the partnership approach, fostering its long-term sustainability. By adopting this framework, healthcare institutions can clarify patient partner roles, standardize partnership practices, and promote AP acceptance within clinical teams. Correspondingly, APs interviewed in this study stressed the importance of communicating their role to clinicians, which would encourage clinicians to refer APs to their patients more frequently. In a scoping review of the ethical foundations of hospital peer support programs, our team uncovered additional challenges for AP institutionalization [43]. Lack of remuneration, though widely accepted by APs, may have contributed to a lack of recognition of their importance. Therefore, institutional acknowledgment could also be catalyzed by formalizing the AP role through financial remuneration and the use of institutional email addresses.

## 5. Limitations

This study is one of the first to explore the motivations, perceived impacts, and institutional integration of APs in hospital settings, including a newly established prostate cancer program. As an exploratory investigation, the study has several limitations. First, the qualitative nature of the analysis lends itself to subjectivity. We ensured scientific rigour through continuous meetings between researchers. Even so, researchers may have had varied methods for classifying quotes into themes. Other limitations have to do with the sample size. Not all the APs were available to participate in interviews, introducing volunteer bias. Nevertheless, interviewers reached thematic saturation after 12 interviews. We determined that we had reached data saturation when, during our qualitative analysis of the last few interviews, no new themes emerged. We reached saturation with the seven experienced APs, and no new themes emerged from the data on the inexperienced APs. Variability across different program settings may have further contributed to bias, limiting comparability. Specifically, the study included five healthcare institutions, subject to structural variations across regions. Additionally, the sample was disproportionately composed of APs from the Montreal area, whose perspectives could have been overrepresented. Including APs from multiple cancer programs—namely, prostate, breast, and colorectal—may have also introduced confounding variables. Program-specific comparisons should be prioritized in future analyses.

Another limitation stems from the scope of the participants selected. The present study focuses exclusively on the views of APs. A comprehensive evaluation of the program would require insights from patients, family members, and healthcare professionals. Interviews with a range of professionals, including managers, clinicians, and nurses, are reported in another article [18]. Lastly, some APs interviewed had not yet had the opportunity to begin following patients and responded based on their initial motivations, which may evolve once accompaniment begins. Follow-up interviews could be of interest.

## 6. Conclusions

This article included interviews with 12 accompanying patients (APs). APs are patients with personal experience with cancer who accompany new patients in their journey, helping them navigate the healthcare system and providing psychosocial accompaniment. Since its launch in 2018, the program has been expanded to several new hospitals and wards. Our objective was to build on past assessments of the AP program in Quebec. This study is the first to provide a comprehensive review of APs’ perspectives on their motivations, their integration into the clinical team, the perceived effects of the intervention and suggestions on how to improve the program. It is also the first study to include APs from different cancer programs and facilities, and with varying levels of experience. We found that experienced and inexperienced APs alike were confident in their abilities to help new patients, which motivated them. APs from rural hospitals that had recently introduced the program were inspired by its use in larger hospitals. The APs perceived a need for the program, repeating that health professionals lack the time to meet patients’ emotional needs. Experienced APs saw a positive impact on patient well-being, personal gratification, and the clinical team itself. In response to concerns stated by clinicians in previous studies that APs may provide unsound medical advice to patients, the APs expressed awareness of the ethical boundaries of their role. The APs maintained professional relationships with their patients. However, experienced APs did not feel integrated into the clinical team, despite efforts made by our team through the overarching research project PAROLE-Onco. Newly trained APs also feared that they were not being well integrated. To improve this integration, we urge healthcare facilities to disseminate information about the program to the public and to emphasize its importance to healthcare providers.

## Figures and Tables

**Table 1:** List of participating facilities.

HCO Number	Location	# of APs	Region Covered	# of APs Recruited
F1	Semi-urban	5	Montérégie-Est	3
F2	Urban	7	Montreal	5
F3	Urban	2	Sherbrooke	1
F4	Semi-urban	1	Montérégie-Centre	1
F5	Rural	3	Côte-Nord	2

**Table 2:** Characteristics of accompanying patients.

Facility Number *	Participant Number	Cancer Program	Sex (F: Female/ M: Male)	Age Group (Years)	Educational Level	Family Situation	Occupation	Started Accompaniments
F1	01	Colorectal	F	65–74	High school	Couple with no children at home	Retired	Yes
	02	Colorectal	F	65–74	College	Couple with no children at home	Part-time worker	No
	03	Colorectal	M	65–74	College	Couple with no children at home	Retired	Yes
F2	01	Prostate	M	55–64	University	Couple with children at home	Full-time worker	Yes
	02	Prostate	M	65–74	University	Couple with no children at home	Volunteer	No
	03	Prostate	M	75+	University	Couple with no children at home	Retired	Yes
	04	Prostate	M	75+	University	Couple with no children at home	Retired	Yes
	05	Prostate	M	65–74	University	Couple with children at home	Part-time worker	No
F3	01	Breast	F	55–64	University	Person living alone	Retired	Yes
F4	01	Lung	F	65–74	College	Person living alone	Retired	Yes
F5	01	Breast	F	45–54	University	Couple with no children at home	Full-time worker	No
	02	Lymphoma	F	35–44	University	Couple with children at home	Full-time worker	No

* Refers to the region in which the interviewed accompanying patient practices. F1: Semi-Urban/F2: Urban/F3: Urban/F4: Semi-Urban/F5: Rural.

**Table 3:** Factors impacting AP integration.

Factor	Obstacles to Integration	Proposed Facilitators
Recognition of the AP role	Lack of clarity concerning the exact roles played by APs. Lack of institutionalization of APs.	Information sessions with clinicians to clarify the AP role. Financial remuneration and institutional email addresses for APs.
Training and standardization	Lack of standardized training, causing variations among facilities.	Creating a common training program for all APs.
Communication with the clinical team	Few interactions among APs and health providers.	Integrating APs into team meetings and care decisions.

## Data Availability

The data used in this study is stored and anonymized. The data is not publicly available, but it may be available upon formal request (please contact the corresponding author).

## Supplementary Materials

The following supporting information can be downloaded at https://www.mdpi.com/article/10.3390/curroncol33010011/s1, File S1: Interview guide.
