# Supplementary material for: Role, Resources, and Integration of Accompanying Patients in Oncology: A Qualitative Study from the Accompanying Patient’s Perspective
Source: Curr Oncol. 2025 Dec 24;33(1):11. doi: 10.3390/curroncol33010011 (PMC12840174; doi:10.3390/curroncol33010011)
Supplement: Supplementary file 1 [file curroncol-33-00011-s001.zip › curroncol-4013252-supplementary.pdf]

Individual interview guide
Accompanying patients (AP)
(T0)

PREAMBLE

- To thank the participant for agreeing to answer our questions as part of our study.
- The interview will last approximately 30 to 40 minutes.

Interview rules

- The interview will remain **confidential**. To protect your confidentiality, your name will not appear anywhere and you will remain completely anonymous.
- At any time during our conversation, let me know if you have any **questions** or if you prefer **not to answer** a particular question. You can also decide to **stop the interview** at any time.
- Remember that we want to know what you think and what you feel. So, there are no right or wrong answers.
- Ensure that the participant agrees to the interview being recorded.

[Start recording]

SETTING THE SCENE

Have you ever been involved in a project with patient partners?
Opening question: Please tell us what led you to become an Accompanying Patient (AP**)**.

1. How would you define the role of an AP? What role(s) do you expect to have as an AP?

2. What form do you think the accompaniment will take (in person, by phone, or via Zoom)? Would you consider sharing your personal contact details? If yes, what are the limits to your availability as an AP (e.g., not 24/7)?

3. How do you see your integration into the care team?
Moderator: guide the group to clearly define how the AP will be integrated…

4. What distinguishes you from other members of the care team?

5. In your view, what factors could **facilitate** the integration of APs?
What about confidentiality: medical records, exchanges with the accompanied patient, and information sharing with the care team?

6. In your view, what factors could hinder the integration of APs?
In an ideal world, what specific resources (human, financial, infrastructural, or informational) should the institution allocate for AP integration? Please address legal

and ethical issues (professional secrecy, medical-record confidentiality, closeness with the accompanied patients).

7. In your view, what effects do/will the integration of APs have?
   Moderator: Which dimensions are involved, in your opinion?

   - **Effects on patients:** symptoms/quality of life, adherence, care experience, partnership and activation, use of the health system

   - **Effects on APs:** meaning-making, social usefulness, grief

   - **Effects on the team:** partnership in care, collaborative practices, improvement and knowledge transfer, interest in working with APs

   - **Effects on the organization:** redesign of care pathways

8. Is there anything else you would like to add—other topics you consider important to address or explore?

Thank you very much for your participation.
